# Interplay Between Peripheral and Central Inflammation in Autism Spectrum Disorders: Possible Nutritional and Therapeutic Strategies

**DOI:** 10.3389/fphys.2018.00184

**Published:** 2018-03-07

**Authors:** Claudia Cristiano, Adriano Lama, Francesca Lembo, Maria P. Mollica, Antonio Calignano, Giuseppina Mattace Raso

**Affiliations:** ^1^Department of Pharmacy, School of Medicine, University of Naples Federico II, Naples, Italy; ^2^Department of Biology, University of Naples Federico II, Naples, Italy

**Keywords:** ASDs, inflammation, obesity, gut-brain axis, therapeutics

## Abstract

Pre- and post-natal factors can affect brain development and function, impacting health outcomes with particular relevance to neurodevelopmental diseases, such as autism spectrum disorders (ASDs). Maternal obesity and its associated complications have been related to the increased risk of ASDs in offspring. Indeed, animals exposed to maternal obesity or high fat diets are prone to social communication impairment and repetitive behavior, the hallmarks of autism. During development, fatty acids and sugars, as well as satiety hormones, like insulin and leptin, and inflammatory factors related to obesity-induced low grade inflammation, could play a role in the impairment of neuroendocrine system and brain neuronal circuits regulating behavior in offspring. On the other side, post-natal factors, such as mode of delivery, stress, diet, or antibiotic treatment are associated to a modification of gut microbiota composition, perturbing microbiota-gut-brain axis. Indeed, the interplay between the gastrointestinal tract and the central nervous system not only occurs through neural, hormonal, and immune pathways, but also through microbe-derived metabolic products. The modification of unhealthy perinatal and postnatal environment, manipulation of gut microbiota, nutritional, and dietary interventions could represent possible strategies in preventing or limiting ASDs, through targeting inflammatory process and gut microbiota.

## Introduction

Over the past decades, the frequency of autism diagnoses has been steadily climbing and has increased the interest of the scientific community (Fombonne, 2009; Boyle et al., 2011; Elsabbagh et al., 2012). Together with Asperger syndrome, Pervasive Developmental Disorder-Not Otherwise Specified and childhood disintegrative disorder, autism is one of the so-called autism spectrum disorders (ASDs) according to the Diagnostic and Statistical Manual of Mental Disorders version 5. Generally, symptoms start at the age of 8 months, become reliably diagnosed around 2 or 3 years and then continue during the entire life (Rapin and Tuchman, 2008). ASD is defined by impairments in social communication, repetitive behaviors, and restricted interests. These variety of symptoms and their different severity, indicate the involvement not only of genetic factors (Abrahams and Geschwind, 2010) in its etiology, but also that of environmental factors (Fujiwara et al., 2016), which have different effects across individuals.

Growing evidences suggest that individuals with ASDs present prominent activation of microglia and astrocytes and severe chronic inflammation (Vargas et al., 2005), both in the periphery and in multiple brain areas, characterized by increased levels of tumor necrosis factor (TNF)α (Al-Ayadhi, 2005; Chez et al., 2007), interleukin (IL)-1 (Al-Ayadhi, 2005), IL-6 (Wei et al., 2011), and interferon-γ (El-Ansary and Al-Ayadhi, 2012).

Exposure to detrimental factors during early brain development can impact its structure and function and increases long-term susceptibility to neurodevelopmental disorders (Bale et al., 2010). Obesity during pregnancy has been suggested to induce fetal brain inflammation (Graf et al., 2016), consistently nutritional composition during pregnancy is another impacting factor on brain functions: for example, polyunsaturated fatty acids (PUFAs) deprivation during pregnancy reduced learning and memory in the progeny, that restored its functions when diet was properly supplemented (Lozada et al., 2017).

In addition, recent studies show that ASD is associated with gastrointestinal (GI) ailments and changes in microbiota composition (van De Sande et al., 2014; Rosenfeld, 2015). Thus, several strategies are proposed to reduce food-related effects in ASD, as gluten- and casein-free diet, elimination of complex carbohydrates and their replacement with monosaccharides, and micronutrients intake, all interventions convergent to correct neurogenesis and neuro-network development. In addition, the use of probiotics, prebiotics, and post-biotics to ameliorate the dysbiosis-associated GI and ASD symptoms has been also proposed.

Now, it is noteworthy to better analyze the impact of all these factors on the behavior and physiology of future generations, also thank to the support of the wide range of animal models resembling human pathophysiology of ASDs (Chadman, 2017) and whether efficacious intervention strategies can be optimized. Evidence associating ASDs to these different conditions will be better analyzed in the following sections.

## Maternal obesity and ASDs

Different studies have recently investigated critical factors during maternal pregnancy that may influence and cause the ASD development in the offspring. Among plausible maternal stressors, Zerbo et al. (2013) underline the impact of maternal fever; other studies evidence that viral and bacterial infections together with maternal gut microbiome alteration are associated with the development of ASD in offspring, through the activation of the maternal immune system (Lee et al., 2015; Choi et al., 2016; Mahic et al., 2017).

Moreover, the impact of obesity during pregnancy on cognitive function and development of CNS disorders in offspring has recently been investigated (Veena et al., 2016; Edlow, 2017). In particular, several findings indicate a link between maternal obesity and the increased risk of developing ASD in children (Leonard et al., 2006; Hamlyn et al., 2013).

Epidemiologic studies provide evidence that obesity and its complications are increasingly rising in western countries (Flegal et al., 2012; Ng et al., 2014), and with it, the rates of obesity during pregnancy (Kim et al., 2007; Heslehurst et al., 2010; Gregor et al., 2016).

In particular, obesity is often a comorbidity for some ailments, as diabetes, cancer, and cardiovascular diseases (Guh et al., 2009). It is associated with pre-natal complications, such as gestational diabetes (Solomon et al., 1997), pre-eclampsia (Bodnar et al., 2005), hypertension (Magriples et al., 2013), and placental dysfunction (Hastie and Lappas, 2014), significantly impacting mother health and embryonic and fetal growth. Moreover, maternal obesity could also induce post- partum complications (Huda et al., 2010; Siega-Riz and Gray, 2013; Moussa et al., 2016) and even long-lasting detrimental effects on offspring metabolism, organ and brain development, increasing the risk of neurodevelopmental disorders (Misiak et al., 2012). However, studies investigating this issue have shown divergent and unclear results.

In details, Dodds et al. (2011) found that mothers with a pre-pregnancy weight exceeding 90 kg had an increased possibility of develop autistic children. Accordingly, Krakowiak et al. (2012) demonstrated a clear relationship between obesity in pregnancy and the occurrence of ASD in their offspring. Similar findings were shown by Bilder et al. (2013) and Reynolds et al. (2014), which also linked maternal obesity to a delay in language skills. More recently, this correlation was also found in offspring of obese women gaining excessive weight during pregnancy (Li et al., 2016).

Other studies have shown a weak direct, but strong indirect relationship between maternal obesity and ASD in the offspring, particularly when maternal obesity was associated with a very low birth weight of new-borns which had two-fold risk to be autistic compared to normal weight new-borns (Pinto-Martin et al., 2011; Abel et al., 2013; Moss and Chugani, 2014). Moreover, ASD is also developed in children born prematurely (Limperopoulos et al., 2008; Abel et al., 2013; Moss and Chugani, 2014). These findings suggest that maternal obesity can raise the incidence of many negative elements, which in turn enhance the risk of developing ASD.

Notably, children born from mothers who were underweight appear to be at greater risk as well of developing ASDs (Getz et al., 2016). Last year, in a study on Danish population, Andersen et al. (2017), reported that, the risk of ASD in the offspring was observed both in underweight [body max index (BMI) < 18.5] and obese (30 ≤ BMI < 35) mothers compared to normal weight (18.5 ≤ BMI < 25) ones. Thus, both conditions may be associated with an increased risk for ASD.

However, although these studies find a clear association, Rivera et al. (2015), underline that they suffer of methodological limitations (Krakowiak et al., 2012; Hinkle et al., 2013; Moss and Chugani, 2014), and deficit in statistical analysis (Stein et al., 2006). Accordingly, Gardner et al. (2015) did not report in their study a clear relationship between obesity in pregnancy and the occurrence of ASD. Considering the importance of these findings, it is critical to also examine the mechanisms by which maternal obesity induced changes in offspring behavior and brain (Van Lieshout et al., 2011; Van Lieshout, 2013).

Several findings from human and animal studies indicate that the increase in inflammatory cytokines, and the dysregulation of nutrients (fatty acids, glucose) and hormones (leptin and insulin) occurring in obese mothers are possible events involved in the impaired development of offspring (Ramsay et al., 2002; Challier et al., 2008; Madan et al., 2009). Indeed, crossing the placenta, these factors impact the development of neuronal circuits of the offspring, impairing behavioral phenotype and increasing the risk to develop ASD. Moreover, obesity results in an increased level of many inflammatory markers, as C reactive protein, IL- 6, IL-1β, and TNF-α (Das, 2001). This relationship has been observed in pregnant obese women, leading to endothelial (Stewart et al., 2007), placental dysfunction (Leung and Bryant, 2000; Nordahl et al., 2008), and impaired fetal development contributing to the risk of ASD in children (Meyer et al., 2011).

A recent review from Nuttall (2017) evaluates maternal obesity, along with infection, and toxicant exposure, as an environmental risk factor for the occurrence of ASD resulting from a maternal inflammatory response.

In several animal studies, pups born from obese mothers revealed alterations in brain-derived neurotrophic factor, a reduction in neuronal progenitor cell proliferation, atypical synaptic stability, and a reduction in dendrite length and branching (Tozuka et al., 2009, 2010; Yu et al., 2014; Hatanaka et al., 2017), all factors generally revealed in mouse models of ASD. To date, little is still known about the possible epigenetic mechanisms responsible of the development of cognitive functions in the offspring and most of the work has focused on DNA methylation in rodent models.

Considering the multiple mechanisms and factors behind the maternal obese status possibly involved in the development of ASD in offspring, a direct association between obese mothers and child ASD diagnosis cannot be established.

## Gut-brain axis

The scaffold of the gut-brain axis is represented by the central nervous system (CNS), the autonomic nervous system (both sympathetic and parasympathetic branches), the neuroendocrine and neuroimmune systems, the enteric nervous system (ENS), and the gut microbiota. All these components intercommunicate via the immune system, the vagus nerve, and other host microbe interactions, and influence each other, constituting a complex network. The main neuroendocrine pathway is the hypothalamic-pituitary-adrenal (HPA) axis, activated in response to various physical and psychological stressors. A crucial player in this communication between peripheral signals and the CNS is the gut microbiota (GM), therefore, this interplay was re-named microbiota-gut-brain axis, viewed as a bidirectional communication system between gut microbes and CNS (Cryan and Dinan, 2012). During development, GM together with immune system, co-evolves with brain neural circuits required for social and emotional cognition, indeed CNS neurotransmission (serotonergic system) is disturbed by an alteration of GM (Clarke et al., 2013).

In mice without gut microbiota colonization (germ-free mice), a mild stress lead to a higher release of corticosterone and adrenocorticotrophic hormone (ACTH) compared to mice with common and no pathogen bacteria (specific pathogen free mice). Moreover, the administration of *Bifidobacterium infantis* partially reversed the stress response in a time-dependent manner, showing the critical role of intestinal colonization in normal development of the HPA axis (Sudo et al., 2004).

GM capability to influence the brain activity is based on the production of neuroendocrine hormones and neuroactive compounds that play a pivotal role in shaping cognitive networks underlying social cognition, emotion, and behavior (Dinan et al., 2015). Neurochemicals produced by GM can influence the host via two ways: they can either be taken up from the gut into the portal circulation and absorbed crossing the blood-brain barrier (BBB) to modulate cerebral function or they can directly interact with receptors expressed by the ENS, influencing the brain function through ENS-CNS connection. The host GM modulates several features of basic neurodevelopmental processes: integrity of BBB (Braniste et al., 2014); neurogenesis (hippocampus and amygdala) (Ogbonnaya et al., 2015); maturation and activity of microglia (Matcovitch-Natan et al., 2016); myelination in prefrontal cortex (Hoban et al., 2016); synthesis and expression of neurotrophins (Desbonnet et al., 2015), neurotransmitters (O'mahony et al., 2015), and their receptors (BDNF, NR2B, synaptophysin, PSD-95).

### Gut microbiota in early life

GM can be influenced and modulated in a dynamic and rapid manner by several factors, including gestational age, mode of delivery, feeding, and antibiotics/probiotics exposure. Indeed, GM in new-born is characterized by low diversity and relative prevalence of Proteobacteria and Actinobacteria phyla parallel to brain development. Then the diversity increases and changes with a prevalence of Firmicutes and Bacteroidetes (Eckburg et al., 2005). Indeed, the first key factor influencing GM is the gestational age, in fact pre-term babies had a large interindividual variation in GM diversity compared to full-term ones (Barrett et al., 2013). Indeed, pre-term babies are often delivered by caesarian section, that is another factor contributing to GM composition. Natural delivery leads to a new-born microbial composition resembling mother's vaginal canal, while those infants delivered by C-section have instead a GM akin to that of mother's skin (Dominguez-Bello et al., 2010; Del Chierico et al., 2015; Hill et al., 2017). Beyond from the mode of delivery, pre-term children are more frequently fed due to a functionally immature gut, leading to the dominance of pathogenic bacteria and a lower diversity, accompanied also by reduced production of bacteria-derived metabolites, such as short chain fatty acids (SCFAs) (Arboleya et al., 2012). However, in this case, breast feeding would increase pre-term infant microbiota consistently with the utilization of human milk oligosaccharides and substrates (i.e., Bifidobacterium species). Indeed, in this phase the GM composition could remain stable or shift, due to environmental events, such as fever, antibiotic therapy, and formula feeding (Costello et al., 2012).

Whether and how all these homeostatic modifications in GM in early life can impact brain development and maturation is an open question whose understanding would enable the development of nutritional and therapeutic interventions for the prevention of later neurodevelopmental illness.

### Gut microbiota and ASDs

Beyond the social and psychological pattern, GI symptoms are a common comorbidity in ASD (Adams et al., 2011; Williams et al., 2011; Chaidez et al., 2014; Li et al., 2017), even if the underlying mechanisms are still unclear.

Germ-free rats and mice revealed, compared to specific pathogen free animals, a reduced sociability, an increased anxiety-like behavior and peculiar brain gene expression profiles, alterations of neurophysiology, strongly reinforcing the idea that GM may affect mammalian brain development and subsequent adult behavior (Desbonnet et al., 2014; Stilling et al., 2015). In particular, an increasing body of evidence shows that the microbiota-gut-brain axis could participate in ASD. The use of antibiotics, possibly affecting GM homeostasis, during pregnancy is a potential risk factor for infantile ASD (Atladottir et al., 2010). Improvement in behavioral and GI symptoms of ASD patients was observed following different treatments able to manipulate GM (i.e., antibiotics and probiotics) (Sandler et al., 2000; Parracho et al., 2005). All these findings suggest that GM modification may contribute to improve ASD symptoms and that microbiota assortment could represent the boundary between environmental and genetic risk factors that convey in ASD.

### Gut microbiota dysbiosis in ASD patients

Several human studies identified dysbiosis in ASD patients with respect to neurotypical control subjects. ASD patients exhibited decreased Bacteroidetes/Firmicutes ratio (Tomova et al., 2015). High-throughput sequencing analysis showed a significant reduction in relative abundance of phylum Bacteroidetes in autistic subjects, mostly classified as severe ASDs (Strati et al., 2017). On the contrary, Son et al. (2015) did not find any alteration in GM composition in a study comparing fecal microbiota in ASD children and neurotypical siblings by qPCR. Specific bacterial genera have been reported to be differentially abundant in ASD individuals compared to controls: *Clostridium, Sutterella*, and *Desulfovibrio* species, *Bacteroides vulgatus, Collinsella, Corynebacterium, Dorea*, and *Lactobacillus* were found increased in ASD patients (Parracho et al., 2005; Finegold et al., 2010; Williams et al., 2012; De Angelis et al., 2013; Strati et al., 2017), while *Bifidobacterium, Prevotella, Akkermansia muciniphila, Alistipes, Biophila, Dialister, Parabacteroides*, and *Veillonella* were frequently found reduced in the intestinal microbiota of ASD children compared to control subjects (Finegold et al., 2010; Wang et al., 2011; Kang et al., 2013; Strati et al., 2017). Despite the vast majority of researches indicate a distinctive GM composition in ASD, it is not possible, up to now, to define a gut microbial signature for ASD, because of small scale pre-clinical studies and conflicting results often reported. Homogeneity for enrolment criteria and technologies used for GM definition would greatly help to share microbiome data to find out association between microbial communities and different pathological states.

### Relationship among gut microbiota, immune dysregulation, GI, and behavioral alterations in ASDs

A bulk of knowledge pinpoints a role for GM in immune dysregulation and inflammation in ASD (Depino, 2013). Autistic patients show alterations in circulating and brain cytokines and in inflammatory factors along with abnormal responsiveness to stimulation of peripheral blood monocytes and macrophages, suggesting a prominent role of inflammation in the onset and/or development of the disease (Grigorenko et al., 2008; Careaga and Ashwood, 2012; Hsiao, 2013). Moreover, deficiencies in the integrity of both gut epithelium barrier and BBB have also been reported in ASD individuals (de Magistris et al., 2010; Fiorentino et al., 2016). Thus, one possibility is that bacterial products, metabolites, and antigens able to translocate through the altered GI barrier, induce peripheral immunoreaction; peripheral cytokines can activate the vagal system which in turn regulates CNS activity (Yarandi et al., 2016). It is also possible for cytokines and bacterial compounds to reach the blood stream, cross the BBB and directly signal to CNS (Ashwood et al., 2011), impacting on neuronal plasticity and consequently on mood and behavior. Experiments that clearly show a correlation among microbial dysbiosis, GI alterations, immunity, and behavior have been reported for animal models of ASD. In a mouse model of environmental risk factors, such as *in utero* valproic acid (VPA) exposure, gut inflammation, altered microbiota, and ASD-like behavioral abnormalities in male offspring were reported (de Theije et al., 2014). In mouse models of maternal immune activation (MIA) the offspring displays principal features of ASD, decreased permeability of GI tract, altered serum metabolome and increased levels of IL-6 in the colon. Notably, probiotic administration (*Bacteroides fragilis*) proved to be a key treatment to ameliorate alterations in commensal microbiota, to restore intestinal permeability and cytokine production and to improve behavioral abnormalities (Hsiao, 2013; Hsiao et al., 2013). Beyond behavioral tests, in a recent study, Coretti et al. (2017) also explored GM profile, intestinal function and immunological features of adult male and female BTBR mice, which represent the main animal model of ASD. These authors identified some key genera, such as *Bacteroides, Parabacteroides, Sutterella, Dehalobacterium*, and *Oscillospira*, in GM profile of BTBR mice. In particular, female BTBR showed a strong link among an autistic outcome, an increased expression of TNF-α, of *Parabacteroides* and *Sutterella*, together with a decrease of *Dehalobacterium, Oscillospira*, and unclassified member of TM7, a subgroup of Gram-positive still uncharacterized, also known as *Candidatus Saccharibacteria*. On the other hand, the autistic profile of male BTBR mice was accompanied by an increased presence of unclassified members of Helicobacteriaceae and low expression of anti-inflammatory IL-10. Furthermore, in these mice, low levels of *Dehalobacterium, Ruminococcus*, and *Desulfovibrio* were associated with increased gut permeability (Coretti et al., 2017). Altogether these studies indicate a strong relationship among GM composition, gut integrity, immunological state, and behavior.

### Influence of microbial metabolites on brain functions in ASDs

Due to the capability to cross BBB, the influence of bacteria-produced metabolites, such as short chain fatty acids (SCFAs), free amino acids (FAAs), and phenol compounds (4-ethylphenylsulfate, 4EPS), in neuropsychological disorders has been deeply clarified (Morris et al., 2017). Several studies showed that the administration or high concentrations of different SCFAs modulated the pathological features of ASD in opposite ways. Indeed, high levels of propionic acid (PPA) have been associated to the onset of ASD. Thomas et al. (2012) demonstrated that intracerebroventricular administration of PPA increased the stereotyped and repetitive behaviors in rats, influencing the metabolism of key neurotransmitters, such as dopamine and norepinephrine and epinephrine. Furthermore, autistic children fed with products containing PPA or which are metabolized in this SCFA, showed an increase of autistic behaviors and GI symptoms. The deprivation of these foods or reduction of PPA-producing bacteria induced by antibiotics ameliorated their clinical condition (Mellon et al., 2000). On the other hand, sodium butyrate, another SCFA, showed opposite effects of PPA. In two mice models of ASD, the administration of sodium butyrate was able to improve autistic outline, reducing repetitive movements and increasing sociability interaction (Takuma et al., 2014; Kratsman et al., 2016).

## Diet and behavior

Most children with ASD displaying GI disorders, display malabsorption (Goodwin et al., 1971), maldigestion (Cade et al., 2000), microorganism overgrowth (fungi, bacteria, and virus) (Finegold et al., 2010), and altered intestinal integrity (de Magistris et al., 2010), causing symptoms, such as diarrhea, constipation, bloating, belching, and visibly undigested foods (Klukowski et al., 2015). Indeed, impairment in carbohydrate metabolism could underline some of the GI ailments occurring in some ASD patients, even if their role in the neurological and behavioral traits is unclear.

The impact of nutrition-related factors in the etiology and symptoms of ASD encourages to combine the conventional therapy based on behavioral and pharmacological approach together with an appropriate diet in order to improve gut health and alleviate the disease severity (van De Sande et al., 2014). Therefore, adopting individual diets tailored to disease symptoms, would prevent the onset of GI dyscomfort, taking into account the dietary reference values for food energy and nutrients and food preferences of the patient.

Nutritionists also require constant patient management in ASD mostly when they are obese, overweight or wasted, to correct an inappropriate diet. Anyway, the dietary therapy needs to be accompanied by other strategies to better handle ASD.

Available online at: Reducing intake of certain food products (i.e., gluten-free or casein-free diets) is associated with less incidence of numerous GI disorders. On the other hand, several studies show that nutritional deficiencies of autistic patients are filled with the supplementation of vitamins and minerals, fatty acids ω-3, probiotics, in combination with pharmacological and psychological interventions, even if supplementation interventions show contrasting, but promising results.

### Gluten- and milk protein-free diets

A typical strategy to decrease food related effects in ASD is a gluten-free and casein-free (GFCF) diet.

GFCF diet consists in the elimination of food containing gluten, and products containing gluten trace amounts. This diet also eliminates casein, a protein present in cow milk and dairy products. The absence in this diet of milk and dairy products leads, however, to calcium, phosphate, and vitamin D deficiency. Therefore, nutrition specialists usually recommend soy or rice milk as substitutes for cheese (Kawicka and Regulska-Ilow, 2013).

The interest in GFCF diet is related to the opioid excess theory of ASD. This theory suggested that autistic children present abnormal metabolism of those two proteins (gluten and casein) possibly due to peptidase deficiencies. This alteration may result in excessive opioid activity in the CNS, altering its function. In particular, gluten- or casein-derived peptides are suspected to be involved in ASD, resembling opioid-like molecules (Piwowarczyk et al., 2017), able to interact with opioid receptors expressed both in the CNS and GI tract or with opioid metabolizing enzymes.

Data to assess the effects of GFCF diet are limited since nutritional strategies and outcome considered varied among studies as did standard diet and monitoring of compliance to GFCF regimen. Many reports showed a reduction of several behavioral symptoms in ASD patients (Knivsberg et al., 2002; Patel and Curtis, 2007), but re-introduction of gluten or casein containing products leads to autistic symptom recurrence (Piwowarczyk et al., 2017).

### The specific carbohydrate diet

The specific carbohydrate diet (SCD), introduced by Gotschall (2004), represents a strategy to reduce symptoms of malabsorption able to limit growth of pathogenic gut microbiota in ASD patient.

This diet largely recommends monosaccharides, whose origin is fruit, vegetables, and honey, whereas it removes complex carbohydrates. The formulation of this diet is based on the evidence that autistic patients have an abnormality in carbohydrate digestion and adsorption, causing residual food accumulation that represents a breeding ground for pathogenic intestinal flora (Williams et al., 2011). The components of SCD are meat, vegetables (onions, spinach, peppers, cauliflower, and cabbage), eggs, natural cheeses, nuts (walnuts, brazil nuts, and almond), fresh fruits, soaked lentils, and beans (Gotschall, 2004). Autistic children treated with SCD report an improvement in attitude, increased skills, and responsiveness (Gotschall, 2004).

### Low oxalate diet

The presence of certain substances, such as oxalate, in GI tract can be strongly linked to an impairment of CNS structure and functionality (Levy et al., 2007). Furthermore, Konstantynowicz et al. (2012) demonstrated that ASD patients exhibited high levels of oxalates in plasma and urine, underlining the possible correlation between this molecule and the disease. The nutriments containing oxalate are numerous beetroots, spinach, cocoa, figs, black tea, lemon zest, black grapes, green apples, kiwis, tangerines, strawberries, oats, wheat, berries, millet, cashew nuts, hazelnuts, peanuts, almonds, and blueberries (Marcason, 2006). Given its presence in several foods, the daily intake of oxalate in a typical western diet exceeds the acceptable daily intake (ADI) (1000 mg/day instead of 250 mg/day). Therefore, ASD patients should reduce the intake of these foods to 40–50 mg/day (Jaeger and Robertson, 2004) but, at the same time, they should add dietary supplements containing arginine, vitamins A and E, glucosamine, taurine, glutathione, magnesium, thiamine, citrate, CoA, magnesium, zinc, and calcium.

### Micronutrients

Micronutrients, essential for neurogenesis and neuro-network development (Curtis and Patel, 2008), have been reported to be reduced in serum, hair, or tissues from ASD children, as shown for magnesium (Strambi et al., 2006), zinc, selenium, vitamin A, vitamin D, vitamin B complex, vitamin E, and carnitine (Filipek et al., 2004). This deficit is related to several factors: autistic children often experience significant eating difficulties, specific food selectivity (Williams et al., 2000; Arnold et al., 2003; Herndon et al., 2009), poor digestion (Williams et al., 2011), gut inflammation (Jyonouchi et al., 2001; Bauer et al., 2007), and reduced levels of vitamin-producing microbiota in the intestine (Guarner and Malagelada, 2003; Wang et al., 2011). Therefore, multivitamin and mineral oral supplement are widely recommended interventions for ASD (Golnik and Ireland, 2009), improving ASD symptoms (Adams and Holloway, 2004; Mousain-Bosc et al., 2006).

It has also been reported that during pregnancy a deficiency of folic acid (Schmidt et al., 2012; Suren et al., 2013) and vitamin D (Cannell, 2008; Grant and Soles, 2009), increase the risk for offspring developing ASD.

### Polyunsaturated fatty acids

PUFA, namely arachidonic acid (AA, 20:4 ω-6), eicosapentaenoic acid (EPA, 20:5 ω-3), and docosahexaenoic acid (DHA, 22:6 ω-3), are crucial for brain development and cognitive and memory functions (Das, 2013).

Maternal intake of PUFAs improved memory in the progeny, suggesting that, supplementation of PUFAs needs to start during pregnancy and continue after delivery until brain development is complete in adolescence. In agreement, breastfeeding improves brain growth and development and memory, since breast milk is richer in AA and DHA compared with formula (O'connor et al., 2001; Auestad et al., 2003; Alessandri et al., 2004). These results suggest that availability of adequate amounts of various PUFAs (both ω-6 and ω-3 fatty acids), especially AA and DHA to the fetus and new-born, is recommended (Das, 2003a,b; Das and Fams, 2003). In fact, women with higher intake of PUFAs before and during pregnancy had a reduced risk of having a child with ASD than those with lower PUFA intake (Fujiwara et al., 2016).

Moreover, PUFAs inhibited the production of neurotoxic TNF-α, IL-1, and IL-6, and increased nitric oxide synthesis, inhibiting neuronal apoptosis and facilitating memory improvement and consolidation (Das, 2003a,b, 2010, 2011; Das and Fams, 2003).

Several studies showed that both plasma and red blood cell phospholipid fatty acid composition is altered in autistic patients. In particular, the levels of ω-3 fatty acids, including DHA, were reduced in the erythrocytes or plasma of ASD patients compared to controls (Vancassel et al., 2001; Bell et al., 2004; Wiest et al., 2009; Al-Farsi et al., 2013; Brigandi et al., 2015; Yui et al., 2016), without significant reduction in the total (ω-6) PUFAs, with a significant increase in the (ω-6)/(ω-3) ratio (Vancassel et al., 2001), although other studies did not support these claims (Bu et al., 2006; Bell et al., 2010).

Indeed, it was reported that the supplementation of EPA/DHA (Amminger et al., 2007) or addition of high amounts of AA (Yui et al., 2012) produced a significant beneficial effect in ASD.

Intriguingly, metabolism of PUFAs, involving several cofactors such as antioxidants, minerals, trace elements, and various vitamins seems to be compromised (Das, 1985). AA, EPA, and DHA are precursors to anti-inflammatory bioactive lipids, such as lipoxins, resolvins, and protectins, involved in wound healing, and neuroprotection from various endogenous and exogenous insults (Das, 2013). It has been suggested that in some autistic patients, the metabolism of PUFAs is deficient or abnormal therefore anti-inflammatory lipids are lower, in spite of an increase of proinflammatory cytokines, oxidative stress, an alteration of various neurotransmitters (dopamine, serotonin, catecholamines, and BDNF), leading to ASD onset and progression, despite PUFA administration.

Interestingly, PUFAs are also able to induce an increase in BDNF levels in the brain (Wu et al., 2004; Bousquet et al., 2009), where it regulates neuronal development and plasticity. In ASDs, abnormalities in PUFAs and BDNF are shown, thus suggesting an interplay between these endogenous molecules impacting brain growth and development and cognitive function.

## Manipulation of gut microbiota

### Probiotics and prebiotics

Probiotics, commonly recommended in GI disorders, are living microorganisms considered beneficial for human health, generally belonging to Gram positive taxa, (i.e., *Lactobacillus* and *Bifidobacterium* genus). On the basis of microbiota involvement in ASD etiology, probiotics have been considered as a therapeutic tool able to impact brain development, and behavior. Therefore, the rationale of the use of probiotics, has been to re-establish the healthy equilibrium of GM altered in ASDs. One of the first evidence showing the impact of GM on ASDs has been demonstrated by Sandler et al. (2000). Eight weeks treatment of autistic children with poorly absorbed oral vancomycin was able to induce a regression of autistic behavioral traits, an effect that was suddenly blunted after vancomycin withdrawal. This study performed on 11 children showed the requirement of a long-term antibiotic therapy not feasible for ASD patients. On the other hand, it also underlined the role of Gram-positive bacteria belonging to vancomycin spectrum in ASD etiology and/or development. The possible underlying mechanism of this antibiotic was supposed to be the decline of neurotoxin-producing pathogens, such as *Clostridium*, whose surviving spores to therapy were responsible, after germination, of the recurrence of ASD symptoms. In fact, Linday (2001) suggested as adjunctive treatment to ASD therapy the administration of *Saccharomyces boulardii* for its efficacy in *Clostridium difficile* colitis.

In a double-blind, placebo-controlled, crossover-designed probiotic feeding study, autistic children ranging between 3 and 16 years of age were divided into two groups, one receiving the vehicle (placebo) for 3 weeks and *Lactobacillus* (L.) *plantarum* WCFS1 for the following 3 weeks, and the other receiving first the probiotic and then the placebo (Parracho et al., 2010). Oral administration of *L. plantarum* was efficacious in autistic children compared to placebo group, not only modulating GM composition and GI symptoms, but also improving autistic behavior. Actually, probiotics have been suggested in autistic patients where GI comorbidities are also shown (Golnik and Ireland, 2009), but are not safe in immunocompromised children (Geraghty et al., 2010).

In a prospective study on preterm children treated with *L. reuteri* or *L. rhamnosus* in order to prevent GI colonization by *Candida* species, the authors reported the reduction of *Candida* in stool from treated infants with both probiotics, as well as GI symptoms by *L. rhamnosus* administration; notably, all treated newborns showed lower incidence of poor neurological outcomes compared to untreated control group, evaluated up to 12 months by the Hammersmith Infant Neurological examination (Romeo et al., 2011). The role of *Candida* GI colonization in neurological status of newborns was suggested by the elevated urine level of a metabolite of several *Candida* species, D-arabinitol, in autistic patients (Shaw et al., 2000). Indeed, the oral treatment of autistic children with *Lactobacillus acidophilus* for 2 months reduced D-arabinitol in urine, indicating this approach useful for improving autistic behavior (Kaluzna-Czaplinska and Blaszczyk, 2012). However, while the level of concentration ability and carrying out orders were improved in autistic children, the response to emotions was not varied.

The influence of GM and its metabolites on ASD has been demonstrated by Hsiao et al. (2013) through maternal immune activation (MIA) mouse model. Actually, the dysbiosis accompanying this model induced a modification in the production of bacteria-derived metabolites that, spilled into the bloodstream of the offspring, underlined the neurological anomalies. In fact, the administration of the *Bacteroides fragilis* reduced MIA-induced increase in 4-ethylphenylsulphate in the offspring and was able to restore gut integrity, to modulate GM and to reduce the impairment in socialization and communication, the stereotypies and the sensorimotor dysfunction.

Some improvement in GI symptoms as well as a reduction in the severity of autistic traits were observed in children with ASD after 6 months of supplementation with Delpro (West et al., 2013), that was a probiotic mixture containing *L. acidophilus, L. casei, L. delbrueckii, Bifidobacterium longum*, and *Bifidobacterium bifidum* combined with the immunomodulator Del-Immune V.

GM impact on host neurology has been shown by the correction of the impairment of social behavior of the offspring from high fat diet-fed mothers by co-housing these animals with healthy pups delivered from mothers on standard diet. Moreover, the treatment with *L. reuteri*, which was shown reduced in the offspring, selectively improves social behavior by promoting oxytocin-mediated functions (Buffington et al., 2016).

The efficacy of probiotics on reducing inflammation in autistic children having GI comorbidities was also shown by Russo (2015), even if the type of probiotics administered was not reported. Russo showed that various probiotic-based therapies induced a reduction in myeoloperoxidase and copper level compared to untreated patients (Russo, 2015), suggesting GM modulation and reduced GI inflammation as main mechanisms underlining those effects.

A recent study performed in children to evaluate the impact of probiotic on CNS functions was conceived administering *L. rhamnosus GG* or placebo in the first 6 months of life of infants and evaluating the onset of neuropsychiatric disorders in the following 13 years (Pärtty et al., 2015). The results indicate a reduction in the later onset of attention-deficit/hyperactivity disorder or Asperger syndrome by the probiotic, showing a link between the early GM and development of these disorders, although no single constant microbiota composition component or change was detected. The study by Tomova et al. (2015) showed an imbalance in GM in autistic children, accompanied by GI dysfunction whose severity correlated with that of autistic symptoms. Unfortunately, apart from the restoration of GM in these patients by a probiotic mix of three strains of *Lactobacillus*, two of *Bifidobacterium*, and one of *Streptocossus*, the possible reversal of the autistic behavior was not analyzed. Conversely, the case study by Grossi et al. (2016) strengthened the efficacy of probiotic therapy in ASD core symptoms. Twelve years old children received VSL#3 mixture of 10 probiotics for 4 months, then the patients were monitored for subsequent 4 months. Apart from the reduction of abdominal symptoms, no improvement was shown for the restricted, repetitive behaviors, but for the social affect, indicating probiotics as possible therapeutics for behavioral abnormalities associated with ASD.

A very recent prospective, open-label study on 30 ASD children from 5 to 9 years old, showed that 3 months of treatment with *L. acidophilus, L. rhamnosus*, and *Bifidobacterium longum*, modulated GM, increasing *Lactobacilli* and *Bifidobacteria* levels in the stool, and improved both GI and autistic symptoms (Shaaban et al., 2017).

In conclusion, many of these studies on probiotic efficacy in ASDs had several limitations, among which the sample size, the patient enrollment, and the criteria for diagnosis; even the design was not always planned in order to assess significant evaluation of clinical outcomes or show side effects. A clinical study on 100 autistic preschoolers is currently in progress in order to gather more information on the effects of a probiotic treatment both on GI function and neurophysiological pattern (Santocchi et al., 2016).

Prebiotics are carbohydrates, including inulin and oligosaccharides, and some food ingredients indigestible by the host, able to induce modifications in the composition and activity of GM. A study by Schmidt et al. (2015) showed a reduction of salivary cortisol secretion in healthy volunteers by fructo-oligosaccharides and Bimuno galacto-oligosaccharides treatment, indicating a suppression of neuroendocrine stress response and an increase of attention span, both events impacting ASD patients. Therefore, even if no study has analyzed strictly the effects of prebiotics alone in ASD animal models or patients, several findings strengthen the possible improvement by these treatments, able to correct pathological features common in ASDs.

It has been demonstrated, indeed, that prebiotics can directly or indirectly affect signaling molecules. At CNS level, for instance fructo- and galacto-oligosaccharides are able to restore BDNF at hippocampal level (Savignac et al., 2013), probably through the involvement of gut hormones. Moreover, prebiotics do not have the survival problem in the GI tract that probiotics do.

### Fecal microbiota transplantation

Fecal microbiota transplantation (FMT) is a procedure through which patients receive the fecal microbiota from healthy donors. Generally, this practice is beneficial in ailments where dysbiosis plays a pivotal role, since it aims to restore GM homeostasis. Obviously, GI disorders, such as inflammatory bowel diseases and irritable bowel syndrome, are the most common ailments suggested for this intervention, even if also other disorders, like autoimmune disease and obesity has been tested with beneficial effects (Aroniadis and Brandt, 2013). Clinically controlled studies are needed to assess not only the efficacy, but also the possible limitations of this intervention, i.e., the choice of donors, the dosage and duration of this treatment, the delivery system, and the side effects. Moreover, before FMT, patients need to be pretreated with antibiotics and bowel-cleansing regimens (Krajmalnik-Brown et al., 2015; Toh and Allen-Vercoe, 2015). The mechanisms underlying FMT positive effects are not completely clear: indeed, they could be related to viable microbiota or viruses, and/or to substances contained in the feces, such as bile acids, vitamins, short chain fatty acids. Considering the emerging role of GI dysbiosis in ASD, there is a growing attention on FMT approach in ASD. FMT in autistic children has been described by Aroniadis and Brandt (2013). Therefore, the efficacy of FMT in autistic children has been recently studied in an open label trial (Kang et al., 2017). Even if an improvement of GI symptoms and autistic behaviors was reported, this study showed lacking points, since it was not placebo controlled, blinded, or randomized.

## Conclusions

Here, we have briefly reported how unhealthy perinatal and post-natal environment could be associated to later changes in behaviors, establishing the crucial importance of the bidirectional communications between peripheral signals and brain. Maternal obesity, early life exposure to over-or under-nutrition and dysfunction of the microbiota-gut-brain axis are possibly associated with the onset and progression of ASDs. Therefore, the interplay between nutrition and microbial composition in ASDs patients is worthy of further investigation. Gut homeostasis, as well as neurobehavioral patterns, depend upon microbes for certain key nutrients, in terms of diversity and amount. Therefore, the alterations of GM would lead to an unbalance of metabolite profile, immune deregulation and activation of pathways impacting neurological function. Up to date no treatment is fully successful in treating ASD, however, a combination of complementary interventions and techniques could relieve at least in part behavioral symptoms. Adjuvant therapies for ASDs include tailored diets, and gut microbiota manipulation (i.e., the use of probiotics and prebiotics), in order to correct dysbiosis and restore a healthy conversation between gut and brain. This would partially lessen both gastrointestinal and neurobehavioral symptoms.

## Author contributions

CC, AL, FL, MPM, AC, and GM wrote the paper. CC, MPM, and GM supervised the review editing. GM decided the overall structure of the review, coordinating the working group.

### Conflict of interest statement

The authors declare that the research was conducted in the absence of any commercial or financial relationships that could be construed as a potential conflict of interest.
